# Elucidating the effect of biofertilizers on bacterial diversity in maize rhizosphere soil

**DOI:** 10.1371/journal.pone.0249834

**Published:** 2021-04-23

**Authors:** Junhong Wang, Lei Liu, Xiaoyin Gao, Jianxia Hao, Mengliang Wang

**Affiliations:** 1 Institute of Applied Chemistry, Shanxi University, Taiyuan, Shanxi Province, China; 2 Institute of Biotechnology, Shanxi University, Taiyuan, Shanxi Province, China; Government College University, Faisalabad, PAKISTAN

## Abstract

This study was conducted to investigate the effect of biofertilizers on the structure and diversity of the rhizosphere bacterial community of maize. Different biofertilizers were applied to maize. The physical and chemical properties of rhizosphere soil samples were analyzed and the rhizosphere bacteria were analyzed by 16S amplicon sequencing. The results showed that treatment with *Bacillus licheniformis* and *B*. *amyloliquefaciens* as biofertilizers increased the soil organic matter (SOM), total nitrogen, total phosphorus (TP), available phosphorus (AP), and available potassium (AK) contents, indicating that the plant growth-promoting rhizobacteria in the biofertilizers might help the host plant to produce root exudates that, in return, recruit beneficial communities due to available sugars, amino acids, organic acids, vitamins, and polymers. The rhizosphere of maize treated with *B*. *subtilis* biofertilizer had the highest diversity and richness. However, the rhizosphere treated with the combined bacterial strains had the lowest diversity and richness, which might be due to the directional increase of the abundance of some bacteria with special functions, but the decrease of the overall bacterial community diversity in the soil. The dominant bacterial phyla were *Proteobacteria* (32.2%–34.6%), *Acidobacteria* (15.0%–21.0%), *Actinobacteria* (13.1%–17.2%), and *Gemmatimonadetes* (9.0%–10.8%), and the dominant bacterial species were *Aciditerrimonas ferrireducens* JCM 15389 (4.3%–5.2%), *Gemmatimonas aurantiaca* (3.2%–4.1%), and *Pyrinomonas methylaliphatogenes* (2.1%–4.8%). The significantly enriched bacterial functions were associated with amino acid metabolism, sugar metabolism, and energy metabolism pathways. The results of a redundancy analysis showed that SOM, TP, and AK were the main factors affecting the microbial community structure in the maize rhizosphere. In conclusion, the application of biofertilizers increased the diversity and richness of the bacterial community in the maize rhizosphere soil. However, combined strain treatment was failed and not an ideal strategy due to the lowest abundance and diversity.

## Introduction

The rhizosphere is a narrow soil zone surrounding plant roots that is inhabited by numerous microorganisms. These microorganisms are involved in various complex biological processes, and the rhizosphere is considered to be one of the most dynamic interfaces on the planet [1]. Plant roots regulate the soil pH, increase soil nutrients, and secrete specific compounds to form a rhizosphere bacterial community structure enriched with beneficial microbes [2, 3]. The microbes inhabiting rhizosphere soil are beneficial for the host plants, particularly in terms of nutrient availability, stress resistance, and defense against soil-borne pathogens [4, 5]. Studies have shown that rhizosphere microbes are influenced by soil nutrients, plant species, and the application of fertilizers [6–8]. In terrestrial agro-ecosystems, changes in the composition of soil microorganisms are powerful indicators of soil bioactivity and crop productivity [9, 10]. Therefore, exploring the changes in the structure and function of rhizosphere microbial communities has become a leading area in the study of agro-ecosystems.

Maize (*Zea mays* L.) is important to the agricultural economy. In recent decades, the extensive use of chemical fertilizers, especially nitrogen fertilizers, has reduced soil nutrient availability and led to environmental degradation [11] and imbalances in the soil and microbial community structure, all of which may affect crop yields [12]. A biofertilizer is a substance containing living microorganisms that has been shown to increase soil fertility and crop production and can be used as an alternative to chemical fertilizers [13, 14]. Biofertilizers have been widely used to inhibit soil pathogens and promote plant growth [15]. Many studies have reported that bioorganic fertilizers significantly alter soil microbial communities and increase crop yield [16, 17]. Related studies have also indicated that both inorganic and organic fertilizers can change the composition of soil bacterial communities by altering the physical and chemical properties of the soil or by altering the carbon input of surface and underground plant residues [18–20]. One study showed the highest microbial diversity for soil samples treated with organic fertilizer combined with NPK fertilizer [21]. Plant growth-promoting rhizobacteria (PGPR) are considered potential biofertilizers for improving the growth and yield of agricultural crops [22, 23]. *Bacillus* is among the most abundant genera in many rhizospheres, and some strains have PGPR activity [24]. *Bacillus* strains can release a number of metabolites [25] that have a strong impact on the environment by enhancing nutrient availability from plants. However, the effects of biofertilizers containing *Bacillus* strains on the bacterial community in the rhizosphere soil of maize have not been fully clarified.

In this study, we investigated whether different biofertilizers containing *Bacillus subtilis*, *Bacillus amyloliquefaciens*, *Bacillus licheniformis*, or a combination of these *Bacillus* strains could affect the physical and chemical properties and bacterial composition of maize rhizosphere soil. We hypothesized that the application of biofertilizers could increase the diversity and richness of the bacterial community in maize rhizosphere soil.

## Materials and methods

### Study site

The test site was located at the Experimental Base of the Economic Crops Research Institute of Shanxi Academy of Agricultural Sciences in Fuyang City, Shanxi Province, China (N 37°14, E 111°46), which is 1,414 m above sea level. The site is located in the Loess Plateau, which is in a temperate monsoon climate zone characterized by hot, rainy summers and cold, dry winters. In 2017, the annual average of sunshine in hours was 2,601.3 h, the percentage of sunshine was 59%, and the annual average temperature, precipitation in the city, relative humidity, and frost-free period in days were 12.6°C, 467.2 mm, 61%, and 179 days, respectively. The physical and chemical properties of 0–20 cm of the ploughed soil in the experimental area were as follows: organic matter, 27.89 g/kg; total nitrogen, 0.43 g/kg; total phosphorus, 0.573 g/kg; total potassium, 15.53 g/kg; available potassium, 163.3 mg/kg; and available phosphorus, 82.12 mg/kg.

### Field experiment

The corn variety used in the test was Qiangsheng 388, which was selected by Shanxi Academy of Agricultural Sciences for corn research; the test fertilizer used was “Stanley” slow release blending fertilizer (N:P_2_O_5_:K_2_O = 26:12:10); and the test strains used as biofertilizers were *B*. *subtilis*, *B*. *licheniformis*, and *B*. *amyloliquefaciens*, which were provided by the Biochemical Laboratory of Institute of Applied Chemistry, Shanxi University. The experiment was carried out on May 9, 2017, using a randomized block design and a protective line. There were five treatments: the control, *B*. *subtilis*, *B*. *licheniformis*, *B*. *amyloliquefaciens*, and combined strains treatments, and each treatment was repeated three times. The control group was only treated with fertilizer; the *B*. *subtilis* group was treated with fertilizer + biofertilizer containing *B*. *subtilis*; the *B*. *licheniformis* group was treated with fertilizer + biofertilizer containing *B*. *licheniformis*; the *B*. *amyloliquefaciens* group was treated with fertilizer + biofertilizer containing *B*. *amyloliquefaciens*; and the combined strains group was treated with fertilizer + biofertilizer containing a mixture of all three *Bacillus* strains in equal proportions (33.3% each). Notably, three *Bacillus* strains were compatible, which was confirmed by plate crossing experiments (S1 Fig).

The biofertilizer was mixed with the organic fertilizer (≥45% organic matter) + 10% *Bacillus* strain [the survival rate in biofertilizer ≥2 × 10^10^ colony forming units (CFU)/g], and the final organic matter content was 40.9%. The fertilizer consumption was 1.5 kg of fertilizer and 3 kg of biofertilizer. The area of the plantation was 20.7 m^2^ (length × width: 11.5 m × 1.8 m), and the corn rows and plant spacing were 62 cm and 25 cm, respectively.

### Soil sampling and analysis

Soil samples were obtained on September 10, 2017. Five soil cores were randomly obtained from each plot, with a depth of 0–20 cm, based on the plum pattern sampling method [26]. After artificially shaking the rhizosphere residue from the maize, a brush was used to collect the maize rhizosphere soil attached to the root. Then, the maize soil samples from the same treatment were mixed together. Each sample was divided into two parts; one part was stored at -80°C until DNA analysis, and the other part was placed on a 0.2-mm sieve to air dry for subsequent determination of the physicochemical properties. Soil organic matter (SOM), total nitrogen (TN), total phosphorus (TP), total potassium (TK), available phosphorus (AP), and available potassium (AK) were measured as described by Lu et al. [27].

### DNA isolation, real-time quantitative PCR, and 16S amplicon sequencing

Total DNA was extracted using the PowerSoil DNA Isolation Kit (MO BIO Laboratories, Carlsbad, CA, USA) from 0.25 g of fresh soil according to the manufacturer’s instructions. The quality of the extracted soil DNA was assessed by 0.8% agarose gel electrophoresis, and the concentration of DNA was determined using a Nanodrop (ND-1000; Thermo Fisher Scientific, USA). The DNA was stored at 20°C until use.

Full-length bacterial 16S rRNA gene sequences were amplified using the forward primer 27F (5′-AGAGTTTGATCMTGGCTCAG-3′) and reverse primer 1492R (5′-ACCTTGTTACGACTT-3′), and a sample-specific barcode sequence was added in the second step of PCR. All test samples were analyzed in triplicate. The PCR amplification conditions were as follows: a pre-denaturation step at 98°C for 2 min, followed by denaturation at 98°C for 30 s, annealing at 55°C for 30 s, and extension at 72°C for 90 s for 25/10 cycles (first/second amplification), and a final extension step at 72°C for 5 min. The PCR amplified products were purified using Agencourt AMPure Beads (Beckman Coulter, Indianapolis, IN, USA) and quantified using the Quant-iT PicoGreen dsDNA Assay Kit (Invitrogen, Carlsbad, CA, USA) on a Microplate reader (FLx800, BioTek, Winooski, VT, USA). After quantification, the amplification products were mixed in equal amounts, and a 16S rRNA library was prepared using the TruSeq Nano DNA LT Library Prep Kit (Illumina). Then, the library was sequenced by paired-end sequencing on the Illumina and PacBio platforms at Shanghai Paisen Biotechnology Co., Ltd. The raw sequencing data were deposited in the National Center for Biotechnology Information (NCBI) Sequence ReadArchive (SRA) database under accession number PRJNA596611 (https://www.ncbi.nlm.nih.gov/sra/PRJNA596611).

### Data analysis

The original sequence data were initially processed using PacBio SMRT (version 5.0.1.9585). The sequences were screened at least three times, with a minimum prediction accuracy of 99% (minfullpass = 3, minPredictedAccuacy = 99). The prediction accuracy was 99%, when CCS was defined as the lower threshold of noise. The files generated by the PacBio platform were trimmed to remove any sequences longer than 2,000 bp.

The sequencing data were processed using Microbiological Ecology Quantitative Insight (QIIME, v1.8.0, http://qiime.org/) software as previously described [28]. High-quality sequences were clustered into operational taxonomic units (OTUs) with 97% sequence similarity using UCLUST (V5.1.221) [29]. OTU classification was performed by BLAST searches for the most popular representative sequences in the NCBI 16S ribosomal RNA database [30]. Alpha diversity was determined based on the Chao1 estimator (http://scikitbio.org/docs/latest/generated/generated/skbio.diversity.alpha.chao1.html), Shannon index (http://scikit-bio.org/docs/latest/generated/generated/skbio.diversity.alpha.shannon.html), and abundance-based coverage estimator (ACE, http://scikitbio.org/docs/latest/generated/generated/skbio.diversity.alpha.ace.html) using QIIME and R software (v3.2.0). To investigate the similarity of community structure among different samples, beta-diversity analysis including principal component analysis (PCA) was conducted at the genus level using R software (v3.2.0).

### Prediction of bacterial functions

Phylogenetic Investigation of Communities by Reconstruction of Unobserved States (PICRUSt), which was developed by Curtis Huttenhower’s group at Harvard University, is commonly used to predict the functional content in 16S metagenomic data [31]. In this study, PICRUSt (http://picrust.github.com/picrust/) (version 1.1.3) [32] was used to predict the Kyoto Encyclopedia of Genes and Genomes (KEGG) functional categories present in the soil samples.

### Calculations and statistical analysis

Difference analysis was performed for each fertilization treatment by one-way analysis of variance and Duncan’s multiple trials using SPSS 22.0. Statistical significance was set at 5%. Redundancy analysis (RDA) of soil bacterial abundance and soil properties was performed using Canoco for Windows 4.5, with default parameters.

## Results

### Physical and chemical properties of soil

The physical and chemical properties of the soil samples obtained from the different treatment groups are shown in Table 1. Compared with the control treatment, the *B*. *licheniformis* and *B*. *amyloliquefaciens* treatment groups exhibited increases in SOM, TN, TP, AP, and AK contents, suggesting that the application of these biofertilizers made the soil more fertile and rhizosphere microorganisms had a significant impact on the soil. However, the biofertilizers had no significant effect on TK content (P > 0.05). A possible reason might be that the PGPR in biofertilizers might promote an increase in root exudates and enrichment of beneficial bacteria, which could effectively improve dissolved nutrients levels in the soil. However, the vast majority of TK in the soil is difficult for plants to use, thus biofertilizers had no significant effect on TK content. When compared to the control soil, the AK contents of the *B*. *subtilis*, *B*. *licheniformis*, and *B*. *amyloliquefaciens* treatment groups were significantly higher. Interestingly, the AP and AK contents in the combined strain treatment were the lowest among all the treatments. A possible reason might be that the application of all three strains to the rhizosphere might promote rapid utilization of nutrients in the soil by the host plants, and could enrich the beneficial flora in the rhizosphere soil, thus affecting the dissolution of nutrients in the soil [33–35].

**Table 1:** Physical and chemical properties of the maize rhizome soil samples.

Treatment	SOM (g·kg^-1^)	TN (g·kg^-1^)	TP (g·kg^-1^)	TK (g·kg^-1^)	AP (mg·kg^-1^)	AK (mg·kg^-1^)
Control group	31.98±0.71bc	0.40±0.00c	0.54±0.00c	15.33±0.93a	41.56±1.56c	115.00±10.41c
*B*. *subtilis* group	29.82±2.37c	0.35±0.00d	0.54±0.01c	14.40±1.20a	42.11±2.45c	135.00±13.23b
*B*. *licheniformis* group	34.60±0.82b	0.52±0.01a	0.74±0.00a	14.87±0.73a	77.89±1.58a	136.67±2.89b
*B*. *amyloliquefaciens* group	37.50±1.40a	0.41±0.01b	0.64±0.01b	15.53±0.47a	58.47±4.02b	170.00±10.00a
Compound strains group	38.45±1.41a	0.51±0.00a	0.50±0.00d	15.67±1.00a	31.75±1.82d	85.00±5.00d

Values are means ± standard error (n = 3). Statistical significance was set at p <0.05, which was calculated using Duncan’s multiple range tests. The same letter represents no significant difference. TN: total nitrogen, TP: total phosphorus, TK: total potassium, AP: available phosphorus, AK: available potassium

### Structural variance in the bacterial communities

**Fig 1:**
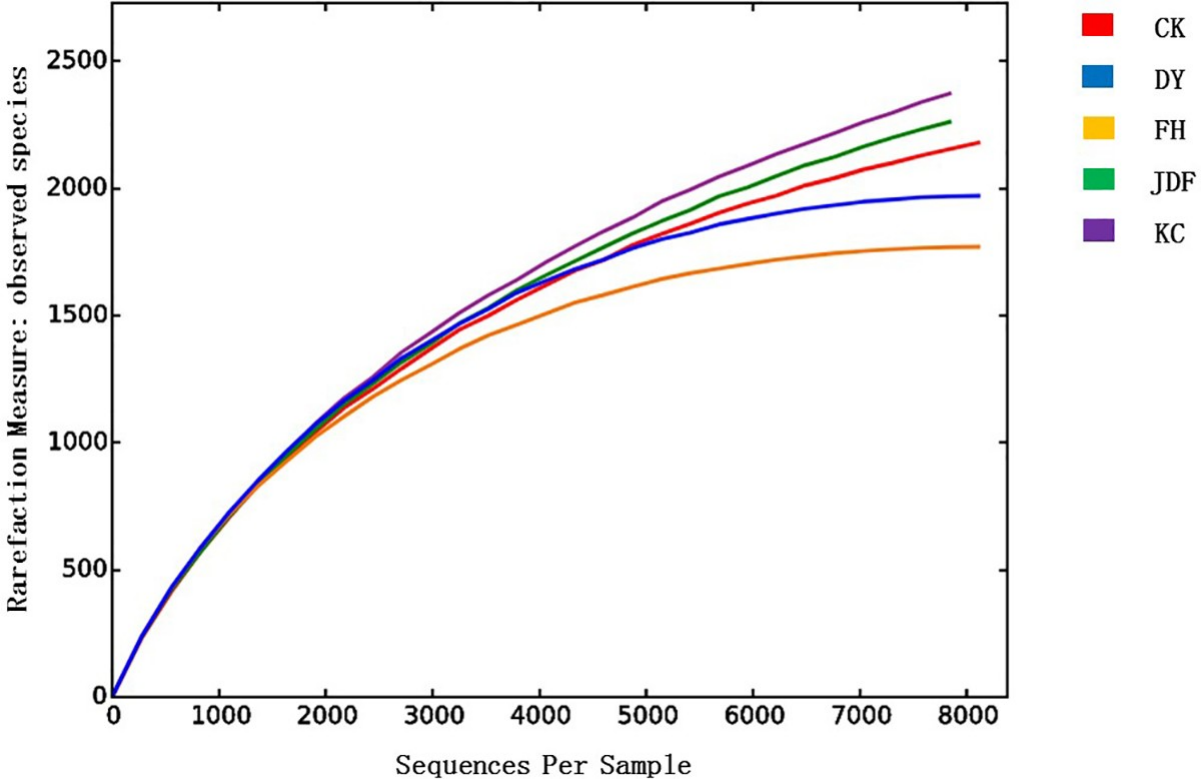
Rarefaction curves based on the observed species in the bacterial communities in maize rhizosphere soil. The abscissa represents the randomly selected sequencing data. The ordinate represents the number of observed operational taxonomical units (OTUs). When the curve tended to be flat, the amount of sequencing data was reasonable. CK, KC, DY, JDF, and FH groups indicate control, *B*. *subtilis*, *B*. *licheniformis*, *B*. *amyloliquefacien*s, and combined strains treatments, respectively.

Using a 97% sequence similarity cutoff, a total of 5,362 OTUs were obtained, which included 24 phyla, 57 classes, 113 orders, 206 families, and 560 genera. The numbers of OTUs in the control, *B*. *subtilis*, *B*. *licheniformis*, *B*. *amyloliquefaciens*, and combined strain groups were 2,180; 2,391; 1,971; 2,275; and 1771, respectively. The results showed that the soil samples from the *B*. *subtilis* group had the highest number of OTUs, at 2,391, while the combined strain group had the lowest number of OTUs at 1,771 (Fig 1). Compared to the control group, the *B*. *subtilis* and *B*. *amyloliquefaciens* treatments increased the abundance of the soil bacterial communities, whereas the *B*. *licheniformis* and combined strain treatments decreased the abundance of soil bacteria.

The Chao1 and ACE indices are widely used to estimate the number of species present in a community. The larger the Chao1 or ACE index, the higher the richness of a community. Unlike the Chao1 and ACE indices, the Shannon index comprehensively considers both the richness and evenness of a community. The higher the Shannon index, the higher the community diversity. As shown in Table 2, among all treatments, the Chao1, ACE, and Shannon indices were the highest in the *B*. *subtilis* treatment, indicating that the abundance and diversity of the bacterial community were highest after *B*. *subtilis* treatment. However, the abundance and diversity of the combined strain treatment were the lowest, as evidenced by the lowest Chao 1, ACE, and Shannon indices. Compared to the *B*. *licheniformis* treatment, the *B*. *subtilis* and *B*. *amyloliquefaciens* treatments increased the richness of soil bacterial community. These data indicate that the different fertilization treatments affect the abundance and diversity of the bacterial community in the soil.

**Table 2:** Sequencing statistics and diversity indices of the bacterial communities in the different soil.

Samples	Sequences number	Chao1	ACE	Shannon
Control group	17506	2592.68	2795.77	10.23
*B*. *subtilis* group	20243	3581.39	3590.80	10.33
*B*. *licheniformis* group	15497	1971.00	1971.34	10.30
*B*. *amyloliquefaciens* group	18735	3148.15	3219.19	10.29
Compound strains group	14268	1771.00	1771.30	10.22

### Species annotation and analyses at different taxonomic levels

#### Comparison of the community composition at the phylum level

**Fig 2:**
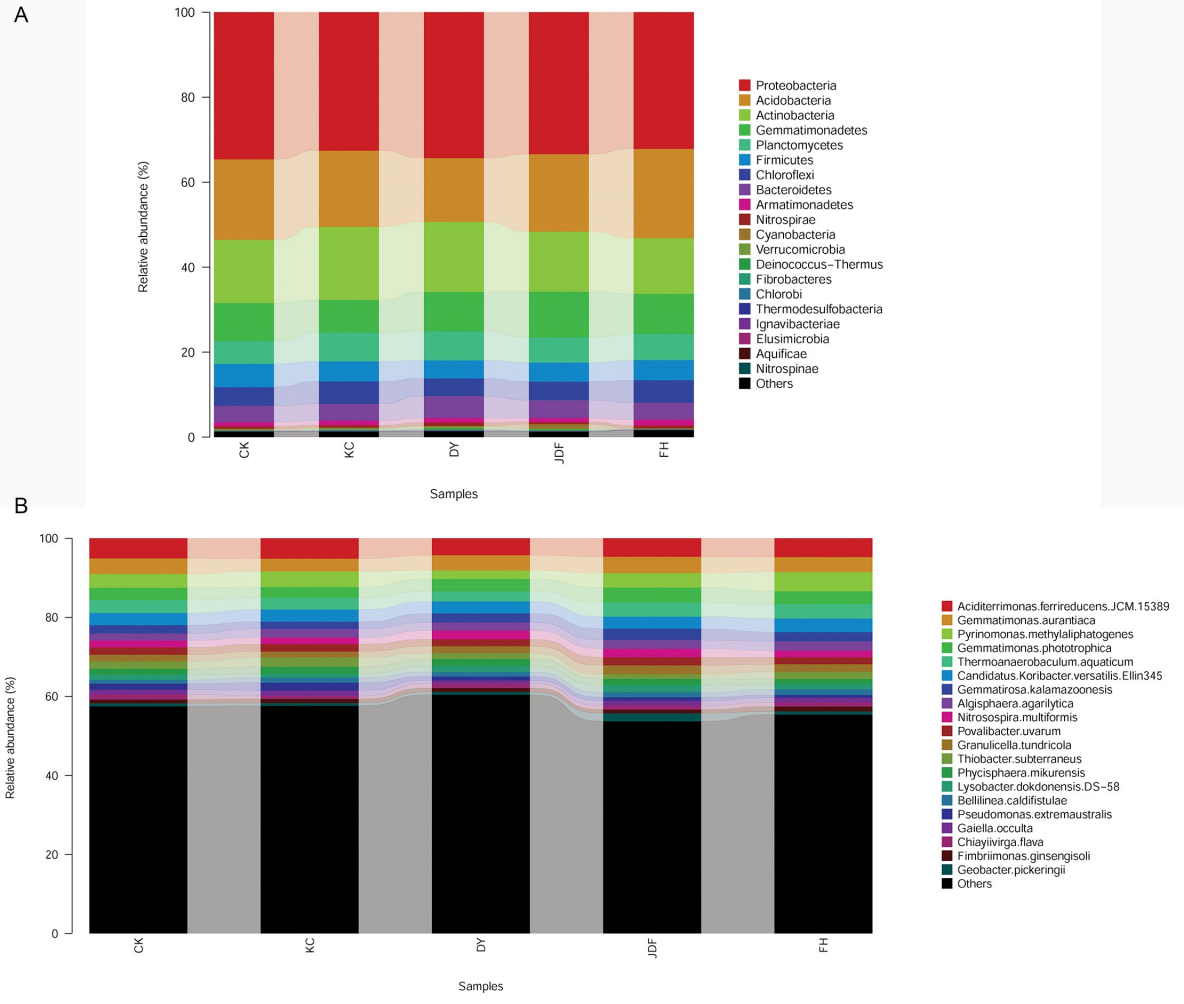
Bacterial community composition at the phylum (A) and species (B) level. CK, KC, DY, JDF, and FH groups indicate control, *B*. *subtilis*, *B*. *licheniformis*, *B*. *amyloliquefacien*s, and combined strains treatments, respectively.

As shown in Fig 2A, 24 phyla were detected, and there was no significant difference among the five treatments. The major phyla were *Proteobacteria*, *Acidobacteria*, *Actinobacteria*, and *Gemmatimonadetes*, which accounted for more than 70% of the bacterial sequences in all treatments, and thus were considered as the four dominant phyla. The relative abundances of *Proteobacteria*, *Acidobacteria*, *Actinobacteria*, and *Gemmatimonadetes* were 32.2%–34.6%, 15.0%–21.0%, 13.1%–17.2%, and 9.0%–10.8%, respectively. The proportions of other bacterial phyla, such as *Planctomycetes*, *Firmicutes*, *Chloroflexi*, *Bacteroidetes*, and *Armatimonadetes*, were relatively low, and thus were considered as non-dominant bacteria.

#### Comparison of the community composition at the species level

Analysis at the species level (Fig 2B) showed that there were 29 species with a relative abundance >1% in the maize rhizosphere soil, and 13 species with >2% relative abundance. The species with the highest abundance in all samples was *Aciditerrimonas ferrireducens* JCM 15389, and its relative abundance in the control, *B*. *subtilis*, *B*. *licheniformis*, *B*. *amyloliquefaciens*, and combined strain groups was 5.1%, 5.2%, 4.3%, 4.7%, and 4.8%, respectively. Other abundant species were *Gemmatimonas aurantiaca*, *Pyrinomonas methylaliphatogenes*, *Gemmatimonas phototrophica*, *Thermoanaerobaculum aquaticum*, and Candidatus *Koribacter versatilis* Ellin345, with relative abundance ranges of 3.2%–4.1%, 2.1%–4.8%, 2.7%–3.8%, 2.6%–3.6%, and 3.0%–3.4%, respectively. Other detected species were present at relatively low abundance, such as *Gemmatirosa kalamazoonesis*, *Nitrosospira multiformis*, *Povalibacter uvarum*, *Granulicella tundricola*, *Thiobacter subterraneus*, and *Phycisphaera mikurensis*.

#### Analysis of differences in the bacterial structure at the genus level

**Fig 3:**
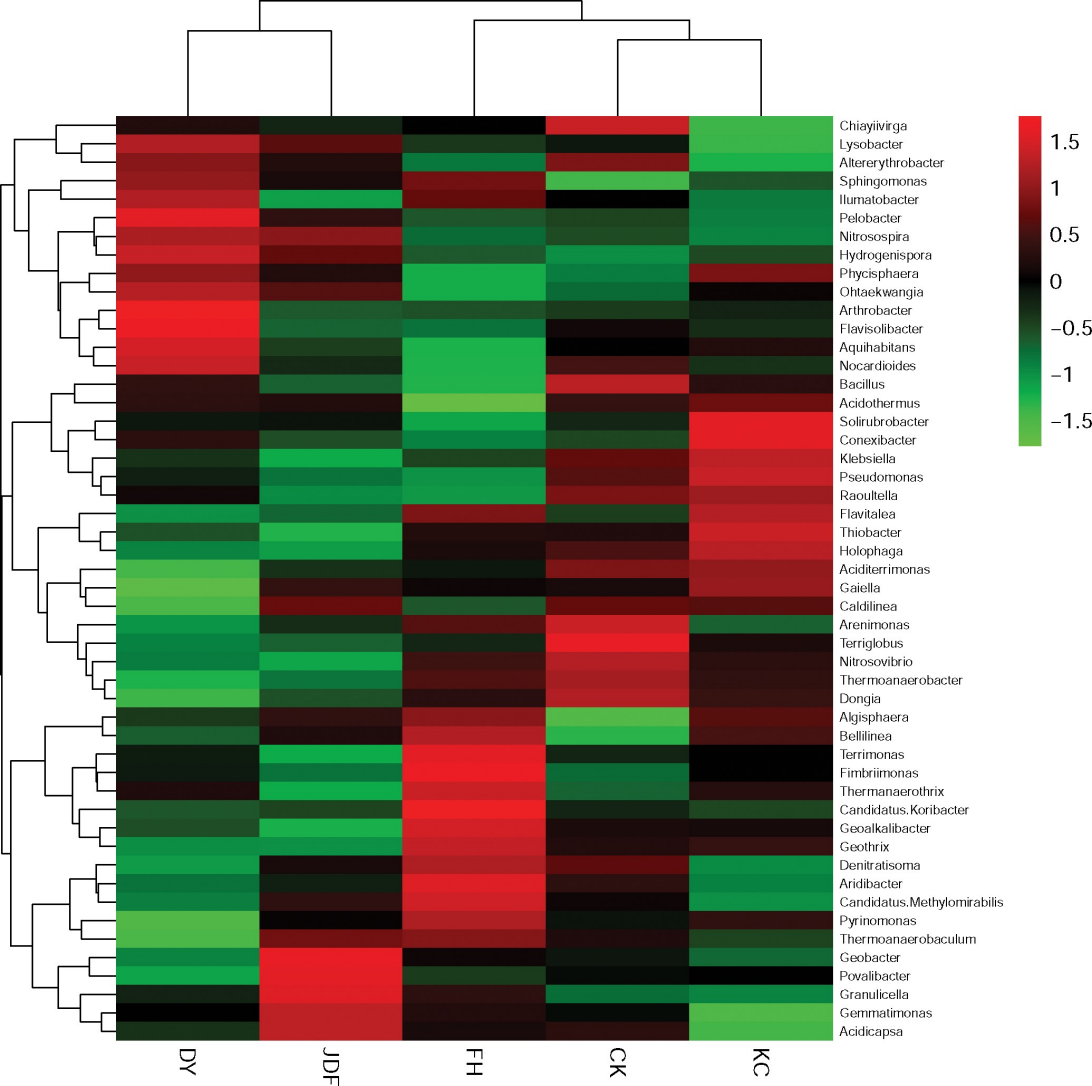
Heat map with a phylogenetic tree generated at the genus level. CK, KC, DY, JDF, and FH groups indicate control, *B*. *subtilis*, *B*. *licheniformis*, *B*. *amyloliquefacien*s, and combined strains treatments, respectively.

To further explore the relationships among the different fertilization treatments and the microbial community structure in the maize rhizosphere soil, the top 50 most abundant genera were clustered and analyzed. A heatmap of the phylogenetic tree (genus level) is shown in Fig 3. Similarity analysis of the communities showed that the *B*. *licheniformis* and *B*. *amyloliquefaciens* treatments were clustered into the same group, while the *B*. *subtilis* and control treatments were clustered into another group. The overall similarity of the microflora structure of the *B*. *subtilis* and control groups was high, while the similarity among the *B*. *licheniformis*, *B*. *amyloliquefaciens*, and combined strain groups was low. Furthermore, PCA was conducted to investigate the similarity of community structure among different samples. The samples in each treatment group were pooled for sequencing and the results of PCA showed that all samples had unique bacterial community structure and they do not share similarities (S2 Fig).

### Prediction of bacterial functions

**Fig 4:**
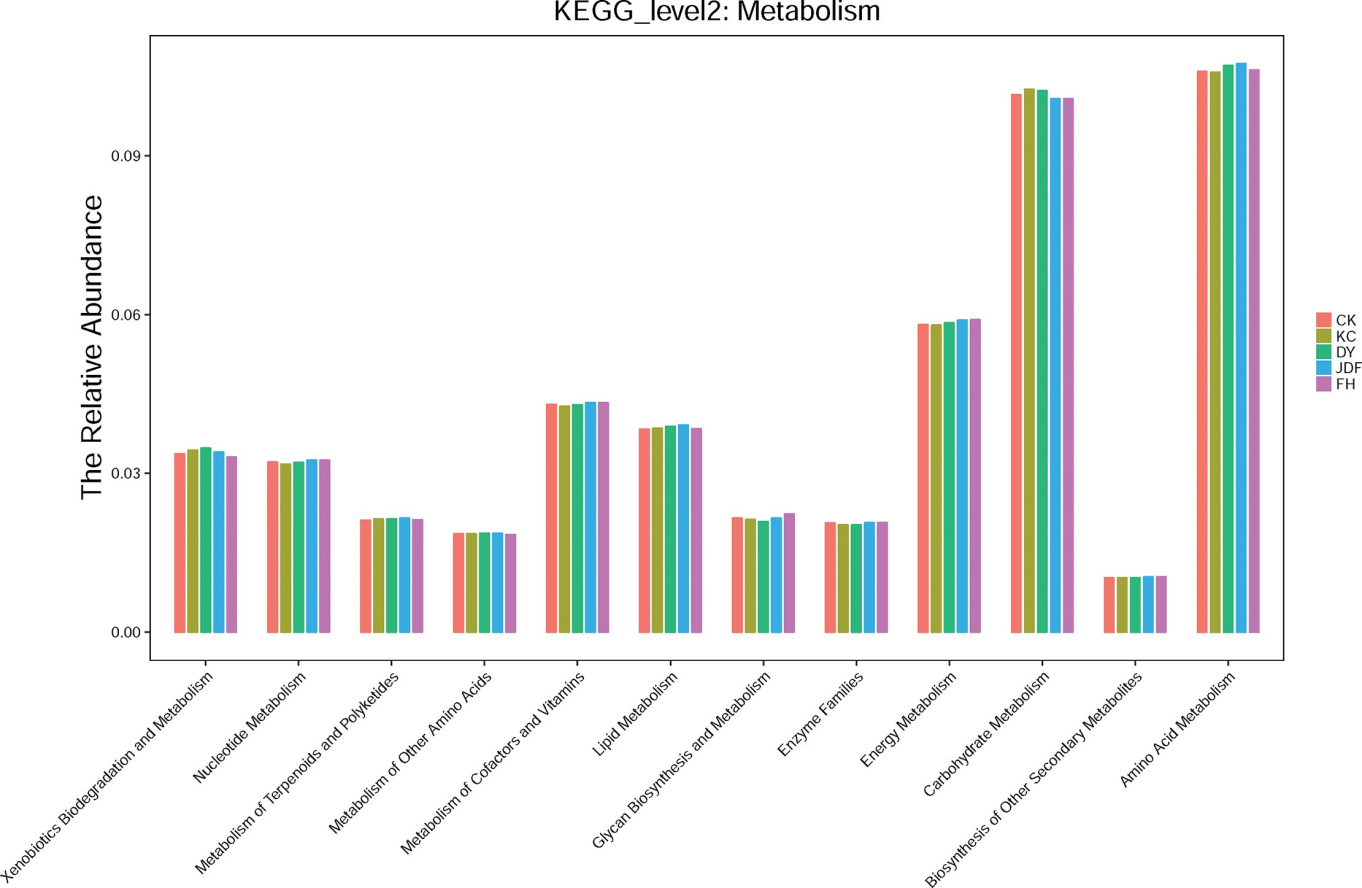
Bacteria functional composition of different soil samples. CK, KC, DY, JDF, and FH groups indicate control, *B*. *subtilis*, *B*. *licheniformis*, *B*. *amyloliquefacien*s, and combined strains treatments, respectively.

A total of 41 second-level KEGG pathways were identified, and 12 metabolism-related pathways were selected for further analysis. As shown in Fig 4, the most abundant pathway in all samples was amino acid metabolism, followed by carbohydrate metabolism, energy metabolism, and metabolism of cofactors and vitamins. However, the relative abundance of the other pathways was lower.

### Relationship between the bacterial community and environmental variables

**Fig 5:**
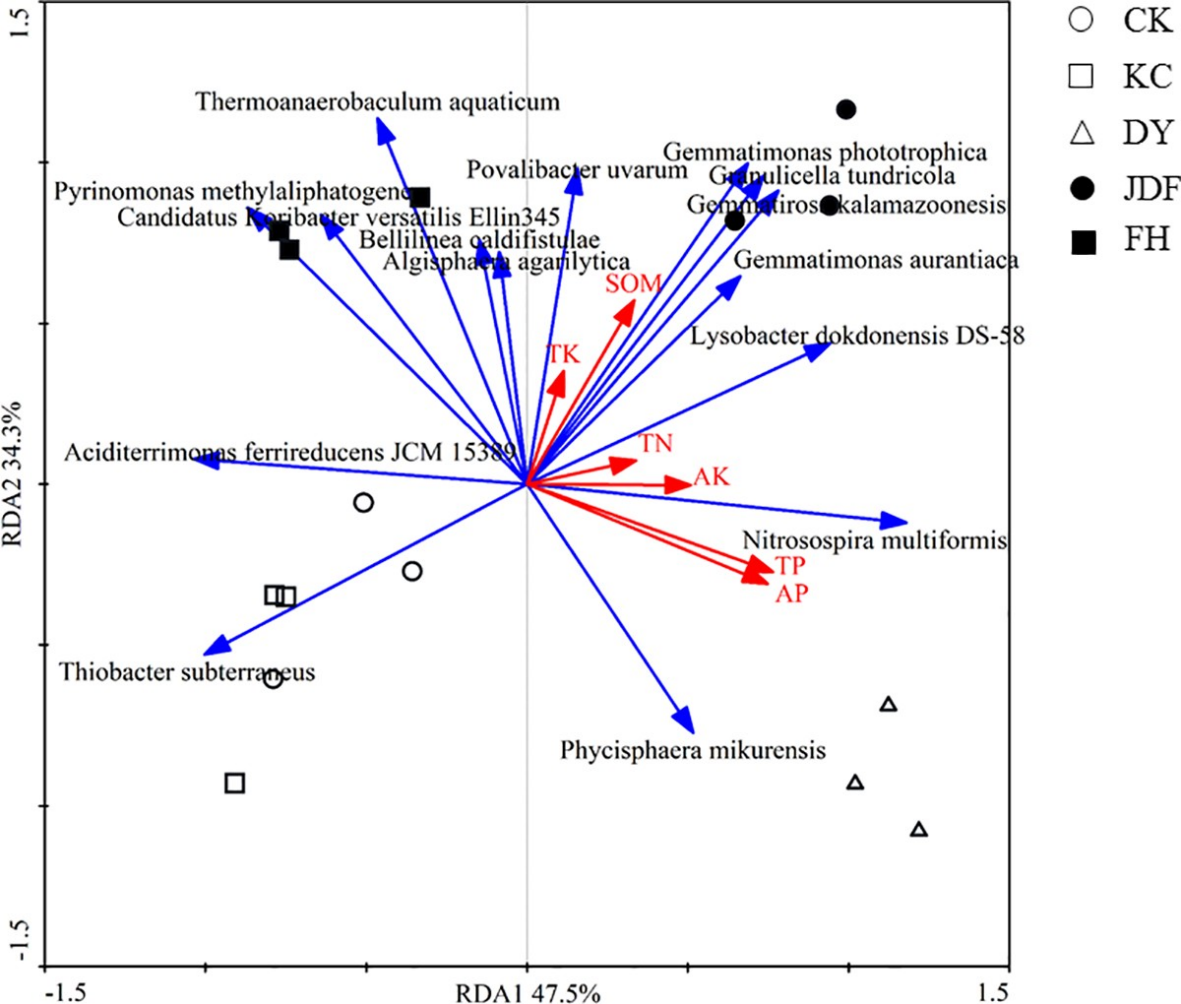
RDA analysis of the correlations between the physiochemical characteristics of the soil and the microbial species in the maize rhizosphere. The blue arrows represent the strains, and the red arrows represent the properties of the soil. The length of the arrows indicates the degree of correlation between the strain and the sample distribution. The angle between the two arrow lines indicates the correlation. The smaller the angle was, the stronger the correlation. CK, KC, DY, JDF, and FH groups indicate control, *B*. *subtilis*, *B*. *licheniformis*, *B*. *amyloliquefacien*s, and combined strains treatments, respectively.

We performed a RDA analysis of the 15 most dominant strains and the physical and chemical properties of the soil. The results showed that all soil properties together accounted for 86.6% of the bacterial community diversity. The first and second components accounted for 47.5% and 34.3% of the total variance, respectively (Fig 5). The rhizosphere bacterial community structure and soil nutrients were correlated, and TP (P = 0.002), AP (P = 0.002), and SOM (P = 0.004) were the main factors affecting the soil bacterial community structure. The dominant species in the soil samples from the control and *B*. *subtilis* groups were *Thiobacter subterraneus* and *Aciditerrimonas ferrireducens* JCM 15389 (Fig 5). *Phycisphaera mikurensis* and *Nitrosospira multiformis* were mainly found in the *B*. *licheniformis* group, and *Gemmatirosa kalamazoonesis*, *Granulicella tundricola*, and *Gemmatimonas phototrophica* were the major species in the *B*. *amyloliquefaciens* group. Samples from the combined strain treatment group mainly contained *Pyrinomonas methylaliphatogenes*, Candidatus *Koribacter versatilis* Ellin345, and *Thermoanaerobaculum aquaticum*.

SOM was negatively correlated with *Thiobacter subterraneus* and *Phycisphaera mikurensis*, but positively correlated with *Gemmatimonas phototrophica*, *Granulicella tundricola*, and *Gemmatirosa kalamazoonesis*. TP and AP were positively correlated with *N*. *multiformis* and *P*. *mikurensis*, but negatively correlated with Candidatus *Koribacter versatilis* Ellin345 and *Aciditerrimonas ferrireducens* JCM 15389. These findings suggest that these strains are related to the transformation, absorption, and utilization of nutrients in the soil.

## Discussion

*B*. *subtilis* and *B*. *amyloliquefaciens* are two important PGPR. *B*. *subtilis* has been shown to improve tolerance to biotic stresses, and colonization of roots by *B*. *subtilis* is beneficial to both the bacterium and the host plant [36]. *B*. *amyloliquefaciens* produces secondary metabolites that are antagonistic to several soil-borne pathogens and is shown to alter both the rhizosphere microbial community and the growth of maize [37]. *B*. *licheniformis* can function as a fungal antagonist and a promoter of plant growth and abiotic stress tolerance [38]. These findings indicate that these *Bacillus* strains exhibit plant-beneficial characteristics. Nevertheless, there is limited research about the effects of biofertilizers containing *Bacillus* strains on the bacterial community in the rhizosphere soil of maize.

In the present study, we found that the application of *B*. *licheniformis* or *B*. *amyloliquefaciens* increased the SOM, TN, TP, AK, and AP contents of the maize rhizosphere soil. These results were consistent with those of previous studies [12, 39]. A possible mechanism by which these biofertilizers increase these nutrients are that the PGPR in the biofertilizers might may help the host plant to produce root exudates that, in return, recruit beneficial bacterial communities, thus increasing the levels of these dissolved nutrients in the soil. It has been previously demonstrated that the organic matter and dissolved organic matter in soil are the main substrates and energy sources for soil microbes [40, 41]. In addition, a growing body of research indicates that the microbial composition is largely determined by environmental factors [42, 43]. Moreover, Ng et al. reported that the chemical nature of soil carbon determines the structure and function of the soil microbial community [44]. Our findings demonstrated that biofertilizer application changed the organic matter in maize rhizosphere soil, which led to changes in the structure and function of the microbial community.

In previous studies, the application of fertilizer (chemical or organic) changed the properties of soil, and these changes were often closely associated with the composition of the soil microbial community [45–47]. Zhou et al. showed similar phenomena, in which inorganic fertilizers decreased the diversity and abundance of bacteria, while organic fertilizers increased the biodiversity of the soil microbial communities [46, 48]. The composition and structure of the soil bacterial community varied among the different treatments. These differences in the soil bacterial communities may be related to the TP, AP, and SOM contents, which are consistent with the available carbon and nutrients and are the main factors shaping the soil microbial community structure [49, 50]. The application of a biofertilizer may promote the enrichment of specific microorganisms in plant roots and attract beneficial microorganisms and promote their proliferation, thereby increasing nutrient availability and resistance to pathogenic infections [51]. The present study showed differences in the composition and structure of the soil bacterial communities of maize treated with biofertilizers or fertilizer. This may be because the fertilization time was too short to have a significant impact on the overall soil microbial community. Moreover, long-term fertilization may result in more stable microbial community changes than short-term fertilization [52, 53]. However, we did not determine the maize yield after treatment due to the short experimental time. Therefore, longer term experiments are required to explore the impact of biofertilizers on plants. Notably, the abundance and diversity of the community in the combined strain treatment soil were the lowest, as shown by the lowest Chao 1, ACE, and Shannon indices. The possible reason for this phenomenon is that the combined strain treatment may result in the directional increase of the abundance of some bacteria with special functions, but the decrease of the overall bacterial community diversity in the soil. Therefore, combined strain treatment was failed and not an ideal strategy. In the future, it is necessary to pay more attention to the study of microorganisms with special functions in the future to understand their role in soil metabolism, and then to clarify the reasons for the low abundance and diversity of soil bacterial communities caused by biofertilizers containing combined strains.

Functional prediction of the sequencing data using PICRUSt showed that the most abundant metabolic pathway in all samples was amino acid metabolism, followed by sugar metabolism and energy metabolism. Amino acids are an essential intermediary in the soil nitrogen cycle and can alter key phenotypes related to symbiotic interactions, plant root growth, microbial colonization, and pathogenesis in the rhizosphere [54]. Thus, we speculate that biofertilizers may alter soil microbial communities and increase crop yield by regulating amino acid metabolism. Moreover, the results showed that the interaction between the soil microbial community and soil chemical properties is mutual. RDA analysis showed the correlation between the microbial community structure and the chemical properties of the soil after different treatments. The levels of TP, AP, and SOM were significantly correlated with the distribution of specific bacteria. Several studies previously reported that microbial growth and activity are affected and limited by SOM and AP [55, 56]. Our results also provided evidence that TP, AP, and SOM are key factors involved in microbial growth in maize rhizosphere soil. Although this study explored the changes in the bacterial community structure in the maize rhizosphere at the molecular level, the pathways and genes of the bacterial strains that affect the metabolism of maize roots will require further study. The rhizosphere community structure is not only affected by the root microenvironment but is also involved a variety of complex effects. Systematic and in-depth research on these issues will provide new pathways to comprehensively understand the bacterial community in the maize rhizome and to identify measures to improve corn yield and quality.

In conclusion, our findings indicated that application of biofertilizer altered the TP, AP, and SOM levels and affected the diversity of the bacterial community in maize rhizosphere soil. This finding provides a theoretical basis for improving the rhizosphere microbial community structure and lays a foundation for further research on rhizosphere functional metabolism. However, given that the abundance and diversity of the community in the combined strain treatment soil were the lowest, combined strain treatment was failed and not an ideal strategy. More studies are still required to explore the combined effects of several strains in the future.

## Supporting information

S1 Fig Plate crossing experiments confirm the compatibility of three *Bacillus* strains. KC, DY, and JDF indicate *B*. *subtilis*, *B*. *licheniformis*, and *B*. *amyloliquefacien*s treatments, respectively.
(TIF)

S2 Fig Principal component analysis (PCA) of the community structure of different samples at the genus level. CK, KC, DY, JDF, and FH groups indicate control, *B*. *subtilis*, *B*. *licheniformis*, *B*. *amyloliquefacien*s, and combined strains treatments, respectively.
(TIF)
