# Supplementary figures and images for: Elucidating the effect of biofertilizers on bacterial diversity in maize rhizosphere soil
Source: PLoS One. 2021 Apr 23;16(4):e0249834. doi: 10.1371/journal.pone.0249834 (PMC8064744; doi:10.1371/journal.pone.0249834)

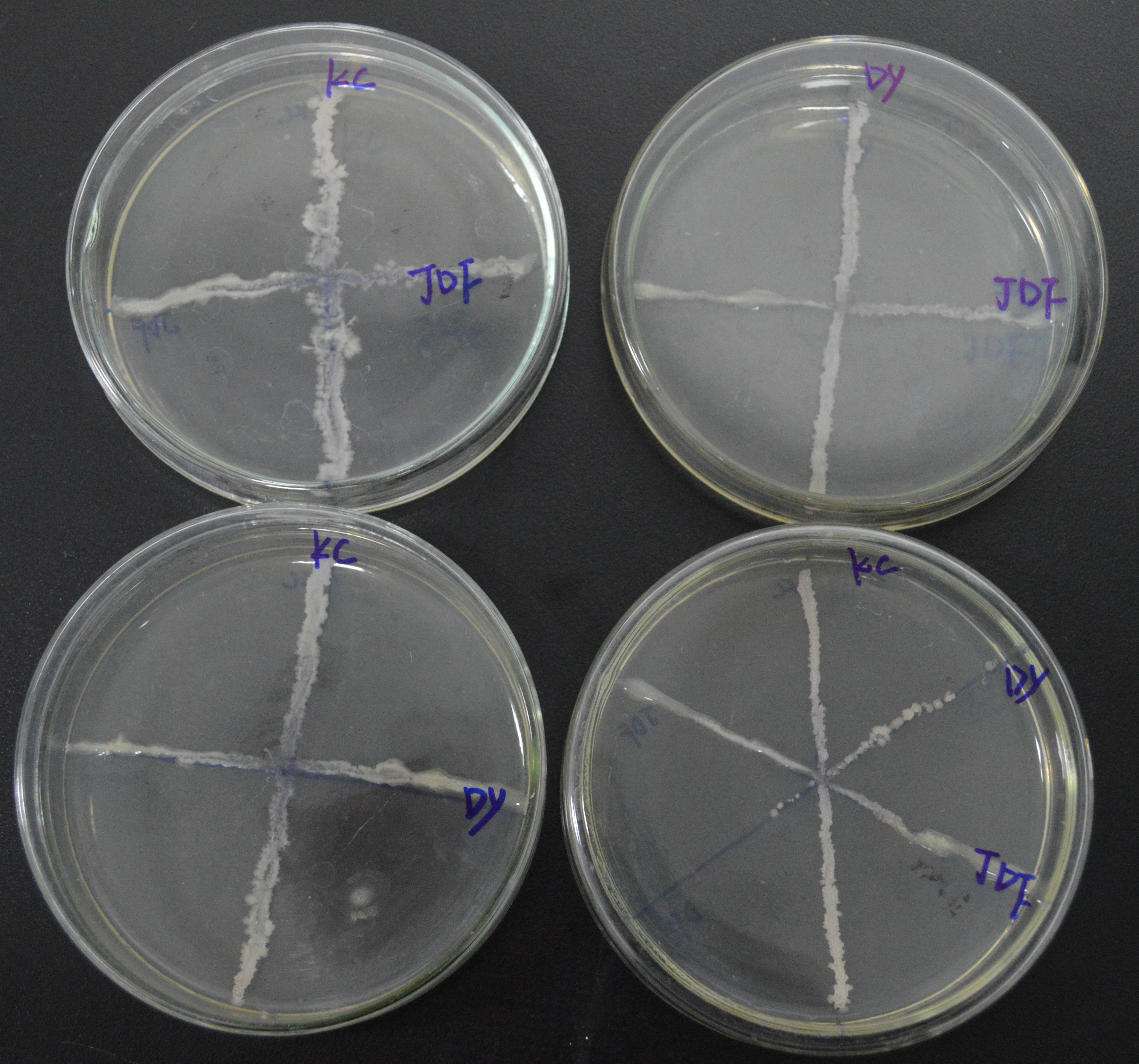

Supplement: S1 Fig — KC, DY, and JDF indicate B. subtilis, B. licheniformis, and B. amyloliquefaciens treatments, respectively. (TIF) [file pone.0249834.s001.tif]

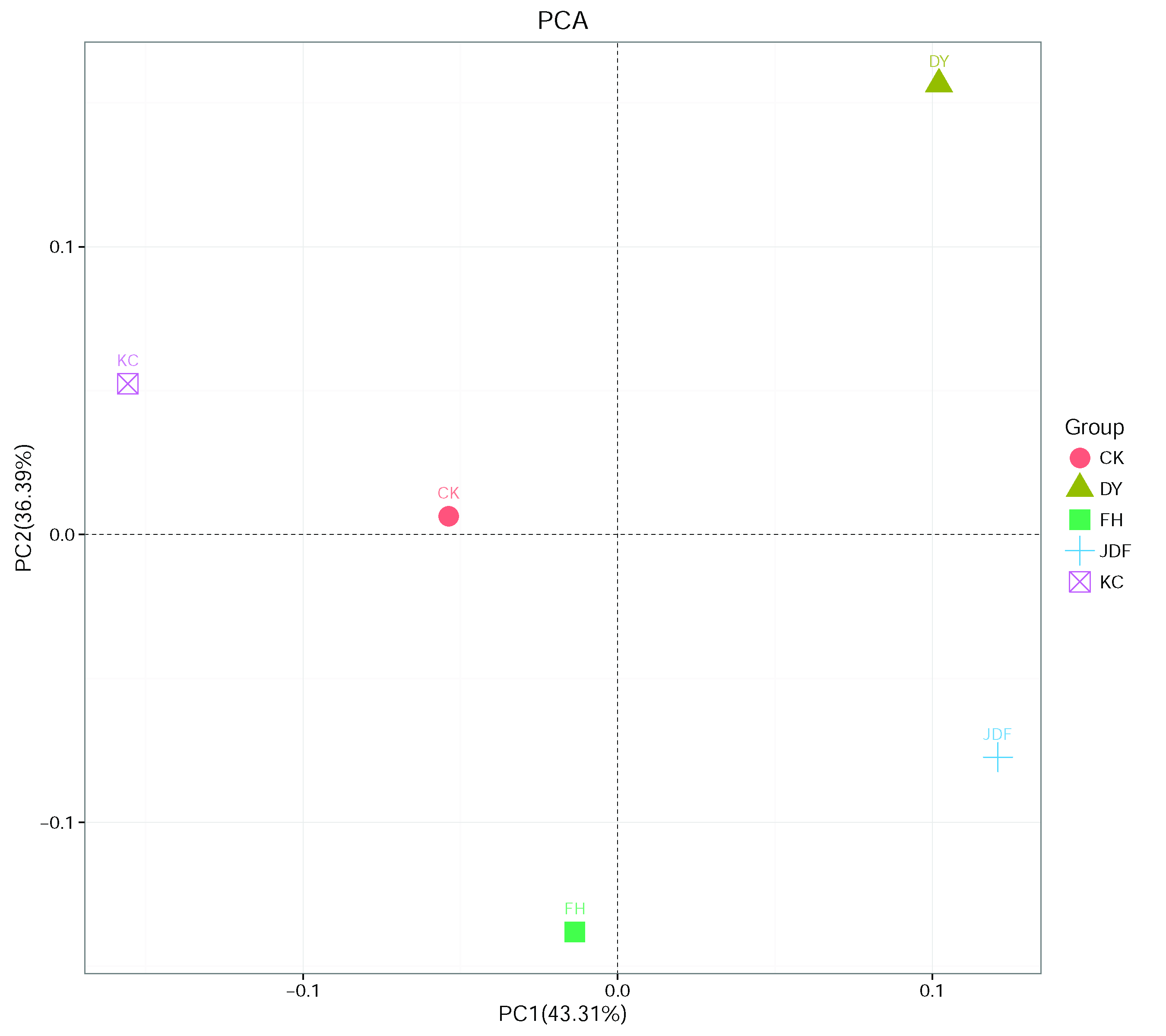

Supplement: S2 Fig — CK, KC, DY, JDF, and FH groups indicate control, B. subtilis, B. licheniformis, B. amyloliquefaciens, and combined strains treatments, respectively. (TIF) [file pone.0249834.s002.tif]
